# Antimongoloid as a pejorative term

**DOI:** 10.3325/cmj.2014.55.77

**Published:** 2014-02

**Authors:** Gwinyai Masukume

To the Editor: As someone with an interest in case reports (1-3), I read your editorial regarding the comeback of the case report with great interest (4). The three case reports that you published (5-7) are a great read. The case report by Nosan et al (6) mentions an “antimongoloid palpebral slant,” which is a term with pejorative connotations (8,9). A more informative and appropriate term would be downward-slanting palpebral fissures (10). One can only hope that with time inappropriate terms will disappear from medical discourse save for historical mention and medical journals should play a very important part in discouraging their use (11).
